# Assisting the Visually Impaired: Obstacle Detection and Warning System by Acoustic Feedback

**DOI:** 10.3390/s121217476

**Published:** 2012-12-17

**Authors:** Alberto Rodríguez, J. Javier Yebes, Pablo F. Alcantarilla, Luis M. Bergasa, Javier Almazán, Andrés Cela

**Affiliations:** 1 Department of Electronics, University of Alcalá, Alcalá de Henares, 28871 Madrid, Spain; E-Mails: alberto.rodrguezfernndez@gmail.com (A.R.); bergasa@depeca.uah.es (L.M.B.); javier.almazan@depeca.uah.es (J.A.); 2 ISIT UMR 6284 CNRS, Université d’Auvergne, 63001 Clermont-Ferrand, France; E-Mail: pablofdezalc@gmail.com; 3 Department of Automation and Industrial Control, National Polytechnic, EC170135 Quito, Ecuador; E-Mail: andres_cela@yahoo.com

**Keywords:** visually impaired, obstacle detection, stereo camera, ground plane estimation, audio warning

## Abstract

The aim of this article is focused on the design of an obstacle detection system for assisting visually impaired people. A dense disparity map is computed from the images of a stereo camera carried by the user. By using the dense disparity map, potential obstacles can be detected in 3D in indoor and outdoor scenarios. A ground plane estimation algorithm based on RANSAC plus filtering techniques allows the robust detection of the ground in every frame. A polar grid representation is proposed to account for the potential obstacles in the scene. The design is completed with acoustic feedback to assist visually impaired users while approaching obstacles. Beep sounds with different frequencies and repetitions inform the user about the presence of obstacles. Audio bone conducting technology is employed to play these sounds without interrupting the visually impaired user from hearing other important sounds from its local environment. A user study participated by four visually impaired volunteers supports the proposed system.

## 1. Introduction

Autonomous navigation is of extreme importance for those who suffer from visual impairment problems. Without a good autonomy, visually impaired people depend on other factors or other people to perform typical daily activities. Within this context, a system that can provide robust and accurate obstacle detection in urban environments, like city or indoors, is much more than desirable. Nevertheless, a major limitation of these systems is the usual distrust of visually impaired community towards the new technologies. As a consequence, this work proposes a user study with visually impaired people in order to obtain relevant feedback information about the system. In addition, the proposed obstacle detection algorithm can be easily integrated into more advanced vision-based localization systems for the visually impaired [1,2].

Nowadays, most of the commercial solutions for visually impaired localization and navigation assistance are based on the Global Positioning System (GPS). However, these solutions are not suitable for the visually impaired community mainly due to low accuracy, signal loss and the impossibility to work on indoor environments. Moreover, GPS cannot provide local information about the obstacles in front of or in the near surroundings of the person. Furthermore, other commercial products available in the market present limited functionalities, have low scientific value and are not widely accepted by the users [3].

Computer vision-based approaches offer substantial advantages with respect to those systems and constitute a promising alternative to address these problems. By means of visual Simultaneous Localization and Mapping (SLAM) techniques [1,4], it is possible to build an incremental map of the environment, providing at the same time the location and spatial orientation of the user within the environment. In addition, compared with other sensory modalities, computer vision can also provide a very rich and valuable perception information of the environment such as obstacle detection [5] or 3D scene understanding [6].

This article presents an obstacle avoidance approach based on stereo vision and a simplistic ground plane estimation algorithm that matches the needs of the visually impaired in their everyday life. Situations like holes on a sidewalk and descending stairs are not specifically addressed in this work. However, the user study demonstrates the effectiveness of the solution that will let the visually impaired achieve a higher level of independence to move in urban scenarios. This system is ears-free because the auditory sense is the most important perceptual source for the blind and it is also hands-free to let blind users hold the white cane.

The rest of the paper is organized as follows. Section 2 reviews the main works on visual navigation, obstacle avoidance and visually impaired applications. Section 3 briefly explains the stereo rig calibration and rectification processes in order to obtain an accurate depth map. The proposed obstacle detection methodology is presented in Section 4 and the acoustic feedback for assisting visually impaired people is explained in Section 5. Experimental results considering challenging environments with many independent moving objects and real blind users are exposed in Section 6. Finally, Section 7 concludes the article and depicts some future works.

## 2. Related Work

In the literature, there have been different approaches related to localization and navigation assistance for visually impaired people that employ different sensory modalities. Each of them has certain advantages and drawbacks. Besides, some approaches propose the fusion of these sensory modalities [7]. The most commonly employed sensors are: GPS, acoustic, radio frequency (RF), laser, vision or the fusion of several of them.

Most of the approaches using GPS information share similar problems: low accuracy in urban-environments, signal loss due to multi-path effect or line-of-sight restrictions due to the presence of buildings or even foliage. The work of Loomis *et al*. [8] states that commercial GPS accuracy considering good satellite visibility conditions is limited to approximately 20 m, which is very high and can represent dangerous situations for visually impaired users when they are walking into unknown or unfamiliar environments. In Differential GPS (DGPS), correction signals from a GPS receiver at a known fixed location are transmitted to the mobile receiver in order to correct its position. However, differential correction requires a separate receiver and the service is not available in many locations. An interesting approach is the one described in [9] where GPS accuracy is improved by adding semantic information into the localization framework to aid visually impaired people in urban environments.

Walker and Lindsey [10] studied the application of spatialized non-speech beacons for navigation of visually impaired users. However, the main disadvantages are the cost of installing and maintaining the network and the limited coverage. A similar drawback presents the work by Kulyukin *et al*. [11], which proposes a robotic system based on Radio Frequency IDentification (RFID) for aiding the navigation of visually impaired users in indoor environments, requiring the design of a dense network of location identifiers.

In [7], a fusion of different sensory modalities is proposed towards a portable indoor localization aid for the visually impaired. In particular, they fused the information from a 2D laser scanner, gyroscopes and a foot-mounted pedometer by means of an Extended Kalman Filter (EKF) framework.

The authors in [5] presented a stereo vision system towards a 6-Degrees of Freedom (DoF) SLAM for the visually impaired. It was used for predicting the next movement of visually impaired users and maintaining a local 3D map of the vicinity of the user. This 3D map information is used to evaluate and detect possible over-head obstacles during the navigation of the users. Pradeep *et al*. [1] described a head-mounted stereo vision system for the visually impaired. By means of stereo visual odometry and feature based metric-topological SLAM, they created a 3D map representation around the vicinity of the user that could be used for obstacle detection and for traversability maps generation. Concerning to obstacle detection, a dense scene flow representation of the environment is proposed in [2]. Dense scene flow [12] is used to detect moving objects or pedestrians in crowded areas improving the performance of standard visual SLAM techniques. This system provides a consistent and improved localization and mapping approach for assisting visually impaired users in highly dynamic environments.

Regarding to obstacle avoidance systems, a very first approach that has been employed in robotics since the early nineties is the Vector Field Histogram (VFH) [13]. This is an obstacle avoidance algorithm that builds a local occupation grid map and bidimensional histograms to represent the obstacles in a Cartesian coordinate system. VFH enhances this approach by the use of a polar grid. The corresponding histogram accounts for the number of feature points measured by a laser sensor. A thresholding is applied to search for free path that allow the navigation of the robot, modeling the relation between the obstacles and the robot as forces. There exist several approaches on reactive obstacle avoidance for local navigation with no prior map that present complex and optimized methods to give linear and angular velocity indications to robotic platforms [14–16]. However, in the case of a visually impaired user, the navigation intelligence and local path planning is provided by the person. Hence, our proposal focuses on the obstacle detection and warning system to assist the handicapped.

The work by Dakopoulos *et al*. [3] shows a comparative survey of wearable devices for aiding the visually impaired. According to it, this article can be framed in the visual substitution category and more specifically, our work lies in the subcategory of Electronic Travel Aids (ETAs). Attending to the employed feedback interface we are interested in related research projects that use acoustic signals to inform the visually impaired. Some of the surveyed systems with audio feedback are based on the echolocation principle and employ several sonar or ultrasonic sensors plus a beeping scheme to warn the user. In the case of vOICe, the images from a camera integrated in the eyeglasses were directly translated into complex sounds that tried to represent the environment, with the disadvantage of an extensive training for the user. Another more advanced and portable-wearable system from the University of Stuttgart built a virtual 3D model of the environment almost in real time, although it was tested only in simulation. The FIU project proposed a non-ergonomic system monitoring six radial directions with six sonars and a PDA, which was tested by four blind-folded users. However, its navigation performance was slow. In relation to systems employing vibro-tactile interface, some of them integrate stereo cameras as sensing modality as proposed here, but they exhibit different pros and cons. In summary, most of the surveyed approaches in [3] are wearable systems embedded in small processing units but they are not validated by visually impaired users and in some cases they lack of experiments in real environments.

On the contrary, the work by Peng *et al*. [17] introduces a monocular approach that employs the embedded camera on a smartphone. This system is capable of detecting on-floor obstacles. The conducted experiments included the participation of five blind users that were aided with vibrations and voice-on-demand from the smartphone. The main disadvantage of this system is that the scenario is simulated despite the fact that some performance tests of the proposed algorithm were done indoors. In addition, it imposes the necessity of a 45 degrees tilt angle of the phone with respect to the ground plane while the user is holding it for seeking possible hazards on the floor. Then, hands-free condition is violated, which is an important matter as stated in [18] by eight visually impaired users.

Other work proposed a complete system with a very low computational complexity [19], but installing a set of striking ultrasound sensors in a jacket. Differently, in another more recent approach [20], the authors put the focus on the obstacle segmentation task, which was based on depth and saliency maps and a neural network. Although, they both yielded processing rates lower than 5 frames per second and they were not tested by visually impaired users.

The main contribution of this system is an obstacle avoidance approach that works in real time and has been tested by visually impaired users. This user study demonstrates the effectiveness of the solution that will let the visually impaired achieve a higher level of independence to move in urban scenarios. This system is ears-free because the auditory sense is the most important perceptual source for the blind and it is also hands-free to let blind users hold the white cane.

## 3. Stereo Rig Calibration and Rectification

**Figure 1.**
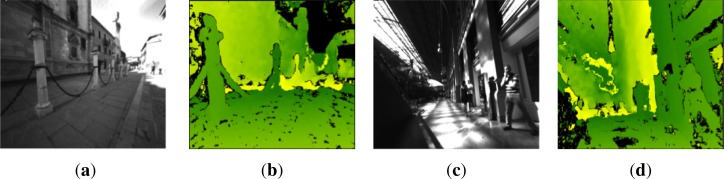
Challenging indoor and outdoor environments: City center of Alcalá de Henares and Atocha railway station, Madrid. (**a**) Captured image Alcalá; (**b**) Disparity map Alcalá; (**c**) Captured image Atocha; (**d**) Disparity map Atocha (Best viewed in color).

Stereo vision systems are commonly used in mobile robotics for localization [2,5], obstacle detection [21] and 3D motion field estimation [22] among others. Considering known stereo calibration parameters (intrinsics and extrinsics) and after distortion correction and stereo rectification, the depth of one 3D point can be determined as:

(1) $$Z=f\cdot \frac{B}{u_{R}-u_{L}}$$

where *u_R_* − *u_L_* is the horizontal disparity computed from the difference in pixels between the horizontal image projections of the same point in the right and left images. *B* is the baseline of the stereo rig and *f* the calibrated focal length. Given the depth of the 3D point *Z*, and the stereo image projections of the same point in both images (*u_L_*, *u_R_*, *v*) (notice that in rectified stereo images *v_L_* = *v_R_* = *v*), the remaining 3D point coordinates with respect to the camera can be determined using the following equations:

(2) $$X=Z\cdot \frac{\left(u_{L}-u_{0}\right)}{f}$$

(3) $$Y=Z\cdot \frac{\left(v-v_{0}\right)}{f}$$

where (*u*_0_, *v*_0_) is the principal point on the left image. Nowadays, there are several algorithms that estimate dense disparity maps in real-time by finding correspondences between the two rectified images of the stereo pair [23–25]. Figure 1 depicts the disparity maps and original left images of indoor and outdoor environments. These are samples of the difficult scenes in real world crowded areas, which pose a strong challenge for the autonomous walking of visually impaired people.

## 4. Obstacles Detection

One of the advantages of stereo over monocular vision is that we can exploit the information from two images at once, obtaining dense disparity maps (between the left and right stereo views at each frame). Since for every pixel that has a valid disparity value we know its 3D position (with respect to the camera coordinate frame) we can use the dense 3D information to detect obstacles in any scenario.

The obstacle detection method described in this section relies on the creation of a virtual cumulative grid, which represents the area of interest ahead of the visually impaired user. In other words, the grid is the area where the potential obstacles have to be detected in order to avoid the user run into them. The goal is to accumulate in the bins of a grid all the 3D points that may belong to obstacles according to its position, depth and height.

### 4.1. Cumulative Polar Grid

**Figure 2.**
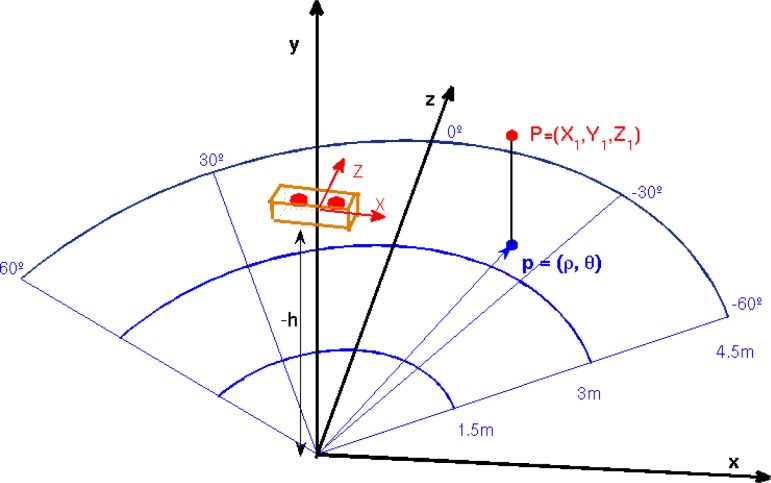
Polar grid. (Best viewed in color).

According to the state-of-the-art, a polar grid is the most appropriate representation for obstacle avoidance in robotics [13]. Similarly, this approach is translated to our problem. Figure 2 depicts the proposed polar grid to accumulate for potential obstacles. A semicircle is defined and subdivided into three radial depths and four angular sections. The farthest distance proposed to detect obstacles is 4.5 m and regions are equally distributed in the fronto-lateral area ahead of the camera.

The three defined depth zones pretend to warn the user with different audio signals about the proximity of the obstacles (side/front walls, pillars, trees, people, vehicles, *etc*.). The maximum bound of 4.5 m and the angular subdivisions correspond to empirical estimations after several experiments with visually impaired users. This estimation also takes into account a fast walking speed of 1 m/s in cluttered environments [26]. Those moving or static objects outside of this grid are not considered as obstacles for the user. Although, given the egomotion of the user and the motion of external objects, they can appear as obstacles upon entering the polar grid zone in posterior frames.

### 4.2. Ground Plane Estimation

**Figure 3.**
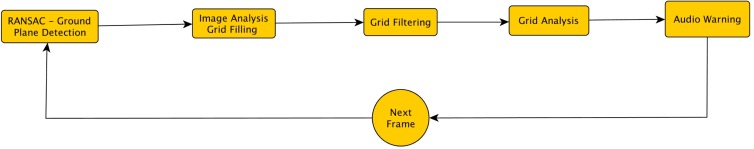
Flowchart of the proposed methodology of obstacle detection and audio warning.

In order to detect obstacles from the 3D position of image pixels, a simple but effective approach is proposed, consisting in the projection of the polar cumulative grid and the 3D point cloud onto the ground plane. The estimation of this plane is performed by a plane fitting algorithm. As a result, the bins of the grid are filled with counts of 3D points of the scene that are above the estimated ground plane. The obtained 2D histogram from the grid is analyzed in order to detect obstacles and inform the audio module to warn the user. The flowchart in Figure 3 displays this process.

The plane fitting algorithm is based on RANdom SAmple Consensus (RANSAC) [27]. It is an iterative method to estimate the parameters of a mathematical model from a set of observed data that contains inliers and is corrupted with outliers. Ground plane estimation is based on the analysis of some 3D points of the scene obtained from the disparity or depth map. Not all the pixels in the image are potentially included in this plane. A thresholding depending on the height of the points is applied in order to reject several pixels of the image. The resultant dataset contains the samples for RANSAC. A different approach is to employ the Iteratively Reweighted Least Squares (IRLS) [28] for the ground estimation. However, in that work, the authors assume that the camera is fixed to a platform, which has fewer degrees of freedom than an application where the user wears the camera. Then, ILRS is a fast method requiring a weight function and a tuning parameter that need more complex heuristics. Hence, we choose RANSAC as a more general and also robust regression method.

The ground plane detection aims at two main factors:

- Firstly, dismiss those pixels that belong to the ground plane as possible obstacles.
- Estimate the height of every pixel of the scene with respect to the ground plane to filter potential obstacles. The objective is to have the ground as a reference plane and to detect obstacles as a function of the pixels height. For example, we are interested in filtering out tree branches that are too high, roofs, banners, *etc*.

The algorithm is basically composed of the following stages:

1. A subset of *N* 3D points are chosen from the thresholded dataset computed from the depth map.
2. Three of these points are randomly selected to solve for the initial parameters A, B, C in Equation (4).
  (4) Ax+By+Cz+D=0
3. The remaining N-3 points are validated against this model to determine the number of inliers that satisfy Equation (5). *λ* is empirically adjusted to 10 cm given the dataset.
  (5) $$D_{i}\left(x,y,z\right)=\frac{\left|Ax+By+Cz+D\right|}{\sqrt{A^{2}+B^{2}+C^{2}}}<\lambda$$
4. A multi-frame filtering approach is employed to ensure the detection of the correct plane hypothesis, taking into account that the ground plane cannot change abruptly between frames. Thus, the distance of the camera to the ground plane is smoothed by an exponential moving average, where the smoothing factor is empirically determined using one video sequence as training.
5. Steps 2, 3 and 4 are repeated several times until convergence. The stopping criteria is given by a maximum number of iterations *T_max_* = 40.

**Figure 4.**
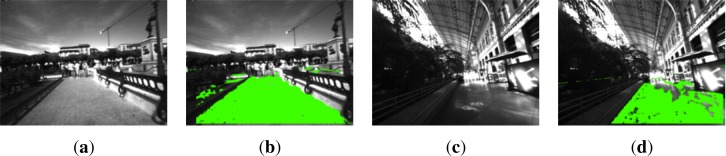
Ground plane detection in two different scenarios: city center of Alcalá de Henares and the Atocha railway station, Madrid. (**a**) Captured image, Alcalá; (**b**) Detected plane, Alcalá; (**c**) Captured image, Atocha; (**d**) Detected plane, Atocha (Best viewed in color).

The images in Figure 4 display some examples of the good performance of the RANSAC algorithm for the ground plane estimation. One of them corresponds to an outdoor scenario in the city center of Alcalá de Henares, while the other is inside the railway station of Atocha, Madrid.

The proposed approach for ground plane estimation works under the assumption that the ground plane is the biggest area in the bottom part of the image. As it can be seen in Figure 4(b), the small vertical wall on the right, corresponding to the back of some benches, is not detected as a valid ground plane. Furthermore, on the left side of Figure 4(d) there is another bench line, where the pixels belonging to it are not considered as ground plane because of their height above the floor.

**Figure 5.**
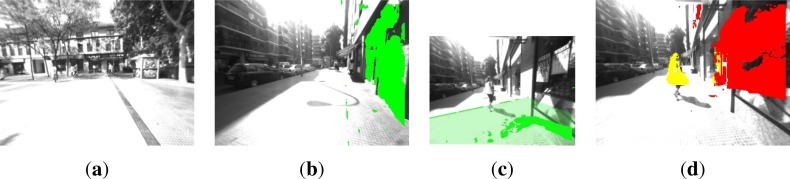
Sample errors in ground plane detection. (**a**) Glossy ground surface due to sun shine. Plane is not detected; (**b**) Detected plane is a wall; (**c**) Wrong ground plane estimation; (**d**) Correctly detected obstacles, but wrong ground plane detection (Figure 5(c)) (Best viewed in color).

The experiments yielded a ground plane detection error in the 11.3% of the frames. This is mainly due to depth estimation errors in glossy surfaces because of sun shine (see Figure 5(a)). There are also a few cases where a vertical plane corresponding to walls is detected as ground plane (see Figure 5(b)). Despite this error rate, obstacles detection is not greatly affected as will be indicated in Section 6. Small planes are still detected in case of too glossy ground surfaces, which leads to the detection of potential hazards for the user above this plane. This situation is depicted in Figure 5(c) and 5(d). Besides, according to the experiments, the detection is robust regardless of the continuous movement of the cameras as the user walked through the environment.

### 4.3. Obstacles in the Polar Grid

**Figure 6.**
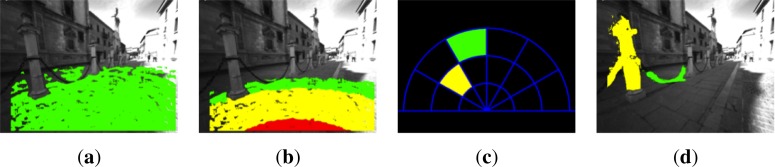
Ground plane and obstacles detection in Alcalá. (**a**) Detected plane; (**b**) Depth zones; (**c**) Detection in polar grid; (**d**) Detected obstacles. (Best viewed in color).

**Figure 7.**
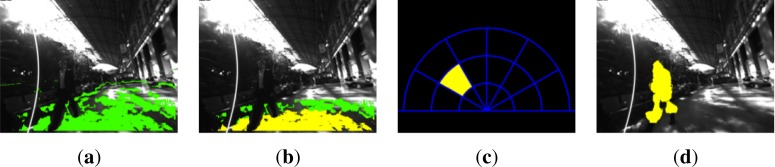
Ground plane and obstacles detection in Atocha. (**a**) Detected plane; (**b**) Depth zones; (**c**) Detection in polar grid; (**d**) Detected obstacles. (Best viewed in color).

Once the ground plane has been estimated, the polar cumulative grid is projected onto it for counting about potential obstacles, as it is depicted in Figures 6(b) and 7(b). In every frame of the video sequence, the entire image is traversed, pixel-by-pixel, discarding those pixels that are not in the grid area or are part of the ground plane. The remaining pixels are checked to determine whether they are candidates to be quantized in one bin of the grid. To do that, the polar coordinates (*ρ_i_*, *ϕ_i_*) of every 3D point projected on the grid are computed, considering the disparity map from the stereo rig. Consequently, the point is assigned to one of the grid bins (see Figure 2) or discarded if it is beyond the upper bound of the grid.

Furthermore, an upper height limit (Y coordinate) is defined in 1.9 m from the ground plane in order to reject obstacles not affecting the visually impaired user, such as tree branches, arcades or small banners. This will prevent the system from continuously warning about objects that are too high. Nevertheless, the value of this threshold can be adjusted in a pre-calibration step depending on the user, such that a very tall user can be warned by the system about these hazards.

If the pixel that is being analyzed satisfies the required conditions, it is counted as a part of a possible obstacle in the corresponding bin of the grid. Finally, some bins are discarded if they are not higher than a certain threshold, which is empirically set to 500 points for 640 × 480 resolution images. These bins are marked as not containing any obstacle. This is carried out to remove false positives caused by erroneous disparity values due to shadows or glows in the images.

Figures 6(c), 6(d), 7(c) and 7(d) depict sample images of the obstacle detection result for pre-recorded sequences in indoor and outdoor environments. Every bin and obstacle associated to them is identified with different colors depending on the warning level for the user. The detected obstacles are over impressed on the original images to match with the information reported in color by the polar grid.

## 5. Audio Warning System

The acoustic feedback is in charge of informing the visually impaired users about the presence of potential hazards in their way. As a consequence, they will react accordingly to avoid obstacle if necessary. There are several approaches in the literature showing a different grade of complexity and usability for the visually impaired [1,3,7,29]. In this work, the ONCE association [30] in Spain has collaborated in the design of this user study, which has been tested by four participants with different grades of visual impairment. According to their experience, audio warning signals would be a good approach to provide obstacle information to them. However, they clearly state the requirement of an audio interface that does not block their ears. Visually impaired people need the sounds from their surroundings in order to infer information for way-finding and detect potential dangerous situations. For example, they use the sounds from cars to understand the orientation of the streets so they can follow a straight trajectory and avoid drifting (when crossing a street there are no landmarks to exploit the white cane for them). Consequently, it is proposed the use of audio bone conducting technology [31,32], which is easy to wear and ears-free.

Beeps are the acoustic signals selected to warn about the relative obstacle position and its proximity. They have been employed in previous works [18,33] too, because they are faster, more effective and less annoying than voice messages. Their codification has been carried out bearing in mind simplicity and the following premises:

1. Two frequencies are employed to differentiate between frontal and lateral obstacles.
2. As beeps can be encoded in stereo, the right, left or both channels are used to notify the obstacles relative orientation. The beep is transmitted to the left channel if the obstacle is on the left side and analogously for the right side. In case of frontal obstacles, the beep is sent to both channels.
3. The proximity of the obstacle is denoted through repetitions of the audio signal. The number of repetitions increases with decreasing distance to the obstacles.
4. The system only warns about the presence of the closest obstacle to the user. However, in case of two lateral obstacles (left and right sides) at the same radial depth in the grid, the system informs about the presence of both. It submits two beeps, with no temporal overlapping, one on each stereo channel. This prevents the user from doing abrupt changes in walking direction, avoiding a possible collision with obstacles on either the right or the left side, e.g., in narrow paths, a wall on the right hand and a person, car or a tree on the other.
5. Table 1 summarizes the acoustic configuration for every grid bin. The letters inside the table correspond to: *f* (beep frequency), *l* (length in ms), *r* (repetitions) and *c* (audio channel).

Audio signals management is performed with the Simple DirectMedia Layer (SDL) [34]. This is a cross-platform, free and open source multimedia library written in C that presents a simple interface to various platforms, graphics, sound, and input devices.

## 6. Experimental Results and Discussion

**Figure 8.**
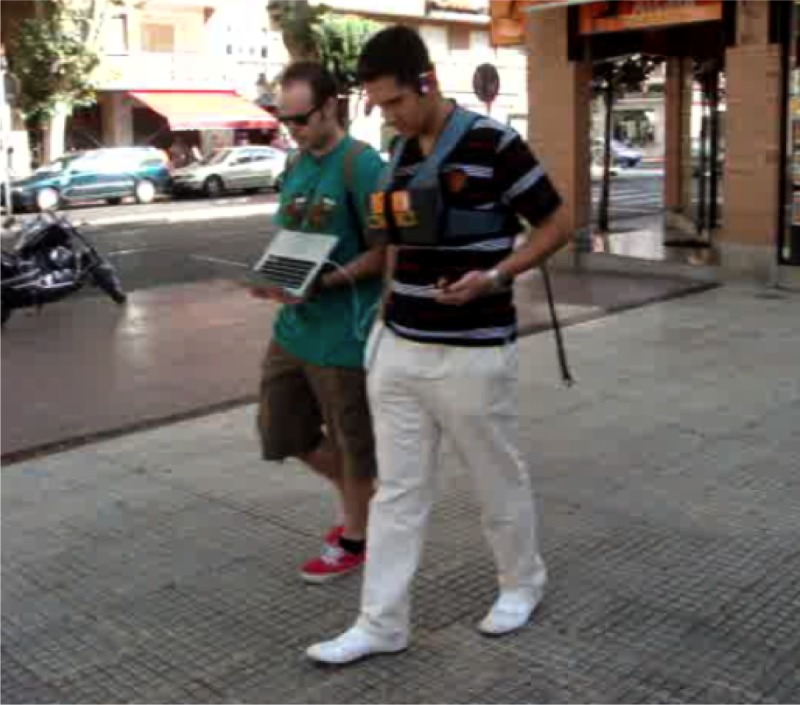
Obstacle detection system for aiding the visually impaired.

The presented vision-based aid system for the visually impaired consists of a stereo camera connected through a Firewire cable to a small laptop for recording and processing the images. The stereo rig is the commercial camera Bumblebee2 [35]. The camera baseline is 12 cm and the practical horizontal field of view is less than 120°. The stereo camera system is attached to the chest of the visually impaired user by means of a non-invasive orthopedic vest, as illustrated in Figure 8.

In the next subsection the conducted experiments are presented, followed by the answers to a questionnaire for the visually impaired participants and concluded with a brief discussion of the obtained results.

### 6.1. Experiments with Pre-Recorded Sequences

**Figure 9.**
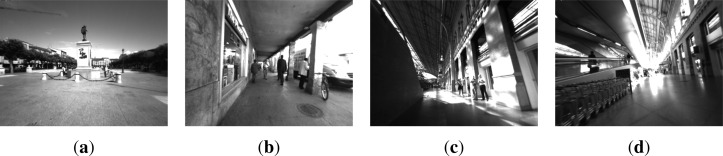
Sample scene images of Alcalá de Henares and Atocha railway station, Madrid. (**a**) Cervantes sq., Alcalá; (**b**) Mayor street, Alcalá; (**c**) Botanic garden, Atocha; (**d**) Main hall, Atocha.

Several tests were carried out to check the performance of the algorithm for the obstacle detection. For this purpose, some video sequences were recorded for large-scale visual SLAM experiments with visually impaired participants. The image resolution was 640 × 480 pixels acquired at 15 frames per second in gray scale. They were captured in highly dynamic environments, with many independently moving objects such as pedestrians or cars. These experiments were conducted inside the Atocha railway station (Madrid, Spain) and in a crowded area of the city center of Alcalá de Henares (Madrid, Spain). In the first set of experiments, we were mainly interested in evaluating the performance of the obstacle detection algorithm without audio feedback. Figure 9 shows some scene images of the routes.

In order to analyze the distribution of the detected obstacles per region, the number of found obstacles on each grid bin is counted. Then, results in Tables 2 and 3 show the number of frames where a bin is activated with respect to the total number of frames. The data corresponds to one of the visually impaired users for each scenario.

As it can be seen in the tables, there are more detected obstacles in the Atocha railway station sequence than in the Alcalá de Henares city center one. This is due to the fact that the station is an important commuting node for passengers. On the other hand, the number of detections is higher in furthest grid bins because, generally, pedestrians tend to avoid coming up against visually impaired people. However, in very crowded areas, like the station, where people move in a hurry and are stressed, there can be some cases where they pass by very close or even bump into handicapped person, as we realized while recording the video sequences.

### 6.2. Experiments with Audio Feedback

After the first set of experiments with the pre-recorded sequences, a new set was conducted employing audio feedback. In this case, two aspects were evaluated: the detection performance of the proposed method and the reliability and usability of the audio signals reporting about detected obstacles position. Four visually impaired volunteers related to the ONCE [30] association agreed to test the system exposed in this work. Only one of them was blind, while the remaining three presented different grades of visual impairment (peripheral view, narrow field of view and extreme light sensitivity). Everyone who participated in the experiments was instructed about the functioning of the system before starting, and a small training in a different area was performed to let them get used to it.

**Figure 10.**
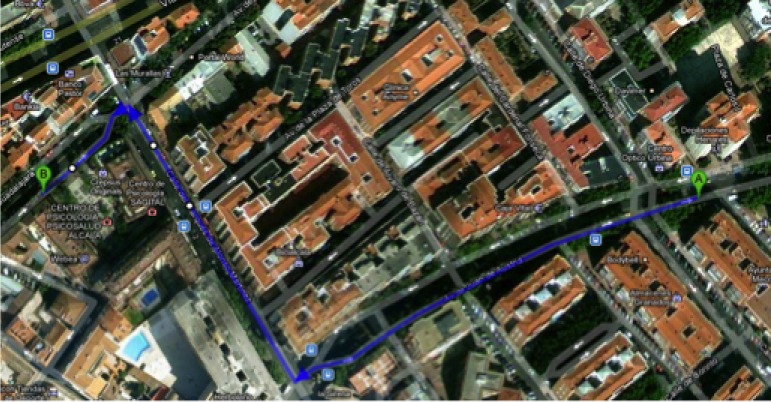
Experiment with visually impaired users. Selected route in Alcalá de Henares (Best viewed in color).

In these experiments, the aim was to test the system in areas in which trees, walking people, cars and in general any obstacles that might endanger the integrity of people with visual impairment may be found. For this reason, we chose a route near the downtown area of Alcalá de Henares, in a commercial area during the mid-evening, so that many people were on the streets. Figure 10 shows the walked path, where the users roamed a total of approximately 800 meters in 15 minutes.

Similarly to pre-recorded experiments, Table 4 exhibits the result of the test for one of the users, in terms of total detection rates on each grid bin.

**Figure 11.**
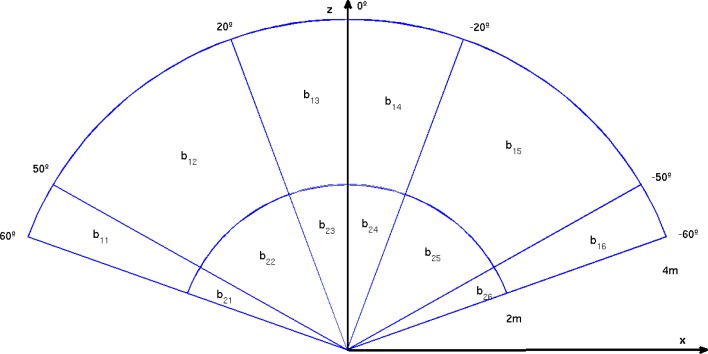
Redesigned polar grid.

Considering the results above and the feedback information from the surveyed visually impaired users, some modifications were proposed for the polar grid. Firstly, a reduction in the number of depth zones from three to two is proposed. Therefore, the new grid is defined with two zones of two meters depth each one. Secondly, more accuracy is required in the orientation of the obstacles with respect to the camera in order to successfully differentiate lateral obstacles from frontal ones. The new angular sections are displayed in Figure 11.

Furthermore, visually impaired users accounted that the presence of a lateral obstacle was not a collision problem, but it allowed them to have a reference for walking, because these obstacles are usually walls. As a consequence, acoustic signals were newly encoded, as shown in Table 5, where the repetitions *r* for lateral bins remains constant for both distances according to the suggestions of the users.

It must be noted that the physical grid deployment in Figure 11 helps to correctly warn the user in case of conflict when different obstacles have been detected. This is illustrated with the following examples:

1. There is an obstacle in *b*_15_ and another in *b*_22_. The user will receive the acoustic signal corresponding to *b*_22_ because it is closer to the user.
2. There is an obstacle in *b*_15_ and another in *b*_11_. The user will receive the acoustic signal corresponding to *b*_15_ because its orientation could represent a future hazard for the user.
3. There is an obstacle in *b*_22_ and another in *b*_25_. The user will receive the acoustic signal on both ears corresponding to both obstacles, because they are at symmetrical bins with respect to the user.

Results in Table 6 demonstrate higher detection rates with the new grid structure and more accurate information in the object angle of approaching. Consequently, the information is more useful for a visually impaired person. They can increase their autonomy especially in outdoor environments where there are a lot of obstacles wandering around the visually impaired person.

**Figure 12.**
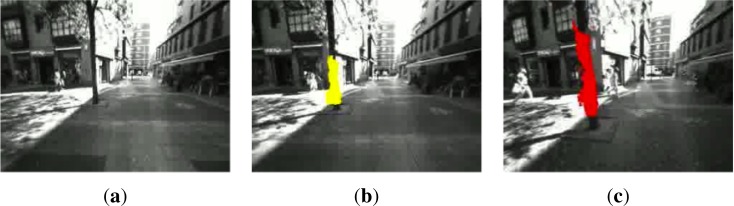
Approaching a tree on the sidewalk. (**a**) Tree in the far distance; (**b**) Tree detected in bin *b*_12_; (**c**) Tree detected in bin *b*_22_ (Best viewed in color).

The next figures display some image samples from these experiments. In particular, Figure 12 shows three frames that are very close in time. The system is able to detect a tree and label it (yellow or red) depending on the distance to the user, who is walking towards the tree. The user modified its trajectory to the right to avoid this obstacle.

**Figure 13.**
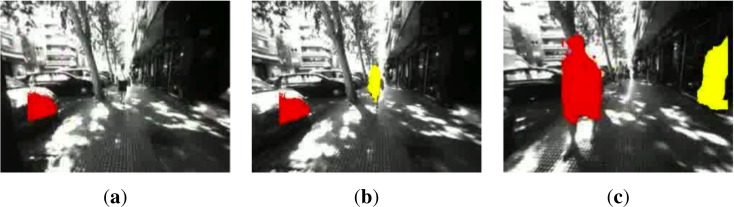
Detection of moving people and static objects. (**a**) Pedestrian in far distance. Car detected in bin *b*_22_; (**b**) Pedestrian detected in bin *b*_13_; (**c**) Pedestrian detected in bins *b*_22_ and wall in *b*_16_ (Best viewed in color).

Similarly, in Figure 13, another person is walking towards the user. Again, the system detects it early enough and the color changes to indicate the potential level of danger for the visually impaired as the obstacle is closer. The user moved to the right to dodge the detected obstacle.

**Figure 14.**
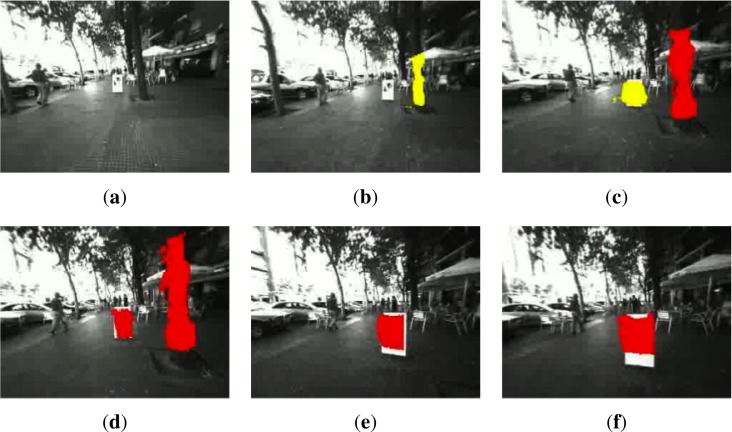
Detection of different obstacles at different depths on a wide sidewalk. (**a**) Obstacles in far distance; (**b**) Tree detected in bin *b*_15_; (**c**) Banner in bin *b*_15_ and tree in bin *b*_25_; (**d**) Banner in bin *b*_25_ and tree in bin *b*_26_; (**e**) Banner detected in bin *b*_25_; (**f**) Banner detected in bin *b*_24_ (Best viewed in color).

Finally, Figure 14 shows six almost consecutive frames where two different obstacles (a tree and a poster advertising) are detected and correctly colored depending on their proximity to the user, who avoids the poster, slightly changing its walking trajectory to the left.

The sequences (images of 320 × 240 pixels) were processed with an Intel Core Duo @ 2.66 GHz and 4 GB of RAM at a frame rate of approximately 11 fps. The RANSAC required a mean value of 45 ms to find the ground plane and the elapsed time for capturing stereo frames was around 37 ms. The remaining time was spent on grid generation and obstacles detection.

Averaging over all sequences, an error rate of 4.19% in obstacle detection and warning is obtained. This value accounts for both false positives and false negatives while informing the user about hazards in its way forward. Typically, the few error cases are due to ground plane estimation error as explained in Section 4.2. Most of the time, the reason comprises depth estimation errors that prevent the system from detecting the obstacles. Moreover, there is 0.67% of frames in which at least one obstacle among several in the scene is erroneously tagged, but the system only provides an acoustic warning of the most important obstacle(s) in the scene. Consequently, these cases can be considered as correct system behavior.

### 6.3. Feedback Information from Participants

After conducting these tests, the involved visually impaired participants completed a questionnaire about their experience with the system. Table 7 summarizes the ratings given by each user for each question that ranges from 0 to 5.

Besides, the following yes/no questions were formulated to the visually impaired users:

- Are the headphones a disadvantage to perceive the other outside sounds?
  All users answered NO.
- Does the system alert you with enough time in advance to avoid an obstacle?
  All users answered YES.
- Is the repetition rate of beeps adequate?
  Users suggested to gradually increase the repetition rate of beeps when increases the proximity of the obstacle instead of using only two depth levels.
- Would you prefer a different audio signal?
  All users answered NO, emphasizing the convenience of the beeps.

### 6.4. Discussion

On one hand, despite the fact that our system is a prototype, it has been tested in real environments with visually impaired users that validate the approach. For the near future, this system could be embedded on smaller and wearable platforms and it is also subjected to improvements like the study of elevation maps proposed in [36], in which a GPU implementation of an MRF stereo framework is presented.

On the other hand, a global detection rate was averaged to evaluate the accuracy of the system obtaining a 95.8% of correct detection and audio warning. However, it is also provided here a maturity analysis as proposed in [3], where 14 features and an overall score are defined as an attempt to quantitatively evaluate the system. Table 8 shows the scores according to the participants answers and the engineers and professors evaluation for every feature defined in the table I of [3].

Every feature corresponds to the following system properties: F1 (real time), F2 (wearable), F3 (portable), F4 (reliable), F5 (low-cost), F6 (friendly), F7 (functionalities), F8 (simple), F9 (robust), F10 (wireless), F11 (performance), F12 (originality), F13 (availability), F14 (future). The first seven features correspond to user’s needs, while the remaining features reflect the developer’s and engineer’s views.

The scores in Table 8 are supported attending to the descriptions provided in Table I of [3] and the numerical results of the experiments before. It must be noted here that some of the features could need further explanation for the reader. The wearability (F2) is supported by the hands and ears-free design, so that the user can perceive and interact with the environment freely, use the white cane, *etc*. However, the presented approach is a prototype that could be integrated in smaller devices in the future, which is precisely the reason of the low portability (F3). Besides, due to the lack of costs information for this future deployment, F5 is not scored. Regarding to the reliability (F4) and the robustness (F9), the system can be affected by wrong plane fitting due to errors in depth estimation or the miss detection of small and too close obstacles due to the inclination and limited field of view of the stereo camera. Finally, the originality (F12) is based on the comments of all the reviewers as well as the overall performance (F11), which also accounts for the total detection rates of the experiments.

Attending to the total score in Table 8 and the comparative results exposed in [3], our system is located in the top 5 ranking. In spite of being a possible subjective measure, it is a good reference for a qualitative comparison over the surveyed works.

## 7. Main Conclusions and Future Work

This work has presented a simple, portable, hands and ears-free system that detects obstacles for assisting visually impaired people. It is an electronic travel aid based on stereo vision and audio feedback. Several experimental results in outdoor and indoor environments with visually impaired participants showed that this system can be very helpful for this user community. Moreover, this system is a module of a larger project for assisting visually impaired people based on visual maps.

Audio bone conducting has been employed during the experiments. It is a non-invasive technology that allows visually impaired users to listen to other important sound sources in the environment (e.g., vehicles) while receiving navigation commands. After the experiments, people who participated in them highlighted some key aspects. First of all, they highlighted the facility to adapt to the system in addition to the great usefulness of the system for a blind person. The users were very satisfied with the performance of the audio feedback. The use of beeps to transmit information about obstacles is not annoying and is preferred over the use of voice messages.

Users also remarked on a number of aspects that can be improved for the future, such as the wearability of the system and the integration of its main components into smaller devices, like microcameras and smartphones. Moreover, they also pointed out on having greater accuracy for the detection of obstacles so that the system is able to warn about the presence of holes and sidewalk curbs, which could be solved by computing elevation maps. On the other hand, it would be also interesting to create a system to replace the beeps by vibrations, which would be useful for a person suffering from blindness and deafness.

Finally, a wider study with more users and further questions about the system are also future guidelines.

## Figures and Tables

**Table 1.** Configuration of acoustic warnings.

	**4,5 – 3 m**	**3 – 1,5 m**	**1,5 – 0 m**
[−60°, −30°]	f = 261, l = 50, r = 1, c = right	f = 261, l = 50, r = 2, c = right	f = 261, l = 50, r = 6, c = right
[−30°, 0°]	f = 2,637, l = 50, r = 1, c = right	f = 2,637, l = 50, r = 2, c = both	f = 2,637, l = 50, r = 6, c = both
[0°, 30°]	f = 2,637, l = 50, r = 1, c = left	f = 2,637, l = 50, r = 2, c = both	f = 2,637, l = 50, r = 6, c = both
[30°, 60°]	f = 261, l = 50, r = 1, c = left	f = 261, l = 50, r = 2, c = left	f = 261, l = 50, r = 6, c = left

**Table 2.** Total detection rates for user 1 in Alcalá de Henares sequence.

	**4.5 – 3m**	**3 – 1.5 m**	**1.5 – 0 m**
[−60°, −30°]	19.1%	11.9%	5.8%
[−30°, 0°]	32.1%	22.6%	2.1%
[0°, 30°]	39.7%	31.2%	6.8%
[30°, 60°]	35.6%	26.1%	3.9%

**Table 3.** Total detection rates for user 2 in Atocha railway station sequence.

	**4.5 – 3 m**	**3 – 1.5 m**	**1.5 – 0 m**
[−60°, −30°]	25.3%	18.6%	5.8%
[−30°, 0°]	44.1%	32.5%	5.4%
[0°, 30°]	46.6%	38.3%	9.1%
[30°, 60°]	42.1%	33.4%	6.2%

**Table 4.** Total detection rates for user 3 in Alcalá.

	**4.5 – 3 m**	**3 – 1.5 m**	**1.5 – 0 m**
[−60°, −30°]	32.5%	20.1%	8.5%
[−30°, 0°]	47.3%	36.7%	6.2%
[0°, 30°]	49.7%	35.8%	5.4%
[30°, 60°]	44.3%	32.1%	11.1%

**Table 5.** Configuration of acoustic warnings corresponding to the deployment in Figure 11.

	**4 – 2 m**	**2 – 0 m**
[−60°, −20°]	f = 261, l = 50, r = 1, c = right	f = 261, l = 50, r = 1, c = right
[−20°, 0°]	f = 2,637, l = 50, r = 1, c = right	f = 2,637, l = 50, r = 4, c = both
[0°, 20°]	f = 2,637, l = 50, r = 1, c = left	f = 2,637, l = 50, r = 4, c = both
[20°, 60°]	f = 261, l = 50, r = 1, c = left	f = 261, l = 50, r = 1, c = left

**Table 6.** Redesigned polar grid. Total detection rates for user 3 in Alcalá.

	**4 – 2 m**	**2 – 0 m**
[−60°, −50°]	18.6%	20.4%
[−50°, −20°]	59.1%	49.8%
[−20°, 0°]	63.2%	54.8%
[0°, 20°]	60.9%	51.2%
[20°, 50°]	53.1%	49.6%
[50°, 60°]	25.7%	23.4%

**Table 7.** Answers of visually impaired participants to the questionnaire.

	**User 1**	**User 2**	**User 3**	**User 4**	**Average Rating**
**How easy is to adapt to the system?**	4	5	5	5	4.75
**How annoying is the vest?**	3	5	4	5	4.25
**How annoying are the headphones?**	0	0	1	1	0.5
**How annoying are the beeps?**	0	0	0	1	0.25
**How useful is the system?**	5	5	4	4	4.5

**Table 8.** Maturity analysis according to [3].

**F1**	**F2**	**F3**	**F4**	**F5**	**F6**	**F7**	**F8**	**F9**	**F10**	**F11**	**F12**	**F13**	**F14**	**Total Score**
9	7	2	6	-	9	6	8	6	-	9	6	10	7	42.15
